# Genomic Selection Integrated with High-Throughput Phenotyping and Speed Breeding for Smart and Greener Rice (*Oryza sativa*) Improvement

**DOI:** 10.3390/genes17080900

**Published:** 2026-07-30

**Authors:** Ha Duc Chu, Trung Quoc Nguyen, Loc Van Nguyen, Nguyen Nguyen Chuong, Quyen Thi Ha, Nguyen Thi Phuong Thao, Touhidur Rahman Anik, Saad Sulieman, Weiqiang Li, Lam-Son Phan Tran

**Affiliations:** 1 Faculty of Agricultural Technology, University of Engineering and Technology, Vietnam National University Hanoi, Hanoi City 100000, Vietnam; quyenht@vnu.edu.vn; 2 VinUni Bigdata Research Institute, VinUniversity, Hanoi City 100000, Vietnam; thao.ntp9@vinuni.edu.vn; 3 Faculty of Biotechnology, Vietnam National University of Agriculture, Hanoi City 100000, Vietnam; nqtrung@vnua.edu.vn; 4 Faculty of Agronomy, Vietnam National University of Agriculture, Hanoi City 100000, Vietnam; nvloc@vnua.edu.vn; 5 Institute of Genomics for Crop Abiotic Stress Tolerance, Department of Plant and Soil Science, Texas Tech University, Lubbock, TX 79409, USA; chuonguy@ttu.edu (N.N.C.); tanik@ttu.edu (T.R.A.); 6 Department of Integrative Agriculture, College of Agriculture and Veterinary Medicine, United Arab Emirates University, Al Ain P.O. Box 15551, Abu Dhabi, United Arab Emirates; saadsulieman@uaeu.ac.ae; 7 State Key Laboratory of Black Soils Conservation and Utilization, Northeast Institute of Geography and Agroecology, Chinese Academy of Sciences, Changchun 130102, China; liweiqiang@iga.ac.cn; 8 Jilin Da’an Agro-Ecosystem National Observation and Research Station, Changchun Jingyuetan Remote Sensing Experiment Station, Northeast Institute of Geography and Agroecology, Chinese Academy of Sciences, Changchun 130102, China

**Keywords:** genomic selection, high-throughput phenotyping, machine learning, speed breeding, rice improvement

## Abstract

**Background**: Rice breeding requires faster development of high-yielding, climate-resilient, resource-efficient, and high-quality cultivars for production systems exposed to environmental variability and increasing input constraints. Genomic selection offers an opportunity to predict breeding value before extensive field evaluation, although its effectiveness depends on the integration of genomic, phenotypic, and environmental information. **Methods**: This narrative review critically examines recent advances in genomic selection for rice and its integration with high-throughput genotyping, high-throughput phenotyping, machine learning, multi-environment prediction, and speed breeding. **Results**: Genome-wide marker data can support early ranking of breeding materials for grain yield, grain quality, disease resistance, drought tolerance, salinity tolerance, and nutrient-use efficiency. Prediction performance is influenced by trait architecture, marker density, training-population size, genetic relatedness between training and candidate populations, phenotypic data quality, and genotype-by-environment interaction. Red-green-blue, multispectral, hyperspectral, thermal, and light detection and ranging platforms can generate temporal traits associated with plant architecture, biomass, water status, nutrient status, and stress responses, which may improve prediction under suitable population and validation designs. Speed-breeding systems shorten generation intervals and facilitate rapid advancement, recurrent selection, and recycling of superior parental lines. **Conclusions**: Integrated breeding pipelines that combine genomic prediction, high-throughput phenotyping, environmental data, and speed breeding can improve selection efficiency and shorten rice improvement cycles. Wider adoption will require affordable technology platforms, standardized data systems, multi-environment validation, breeder capacity development, and collaborative data-sharing frameworks for smart and greener agriculture.

## 1. Introduction

Rice (*Oryza sativa* L.) is a principal food crop for more than half of the global population and supports food security, farm income, and rural employment across Asia, Africa, and other rice-dependent regions [1,2]. Global rice production in 2025/26 is projected to reach 541.3 million metric tonnes on a milled basis [3], while population growth, urbanization, changing diets, and climate-related production risks continue to increase pressure on rice supply systems [2,4]. Conventional rice improvement depends on the identification of parental lines with desirable agronomic traits, planned hybridization, generation advancement through self-pollination or doubled-haploid production, and repeated phenotypic selection from segregating generations to multi-location yield trials [5,6,7]. Breeders evaluate grain yield, days to flowering, plant height, lodging resistance, grain quality, pest and disease resistance, and tolerance to drought, salinity, heat, submergence, or nutrient deficiency across seasons and production environments [6,8]. This process requires extensive land area, labor, seed resources, and several years before superior lines can be selected for regional testing and cultivar release, which has increased interest in genome-wide prediction approaches that permit earlier selection decisions.

Recently, genomic selection (GS) uses genome-wide molecular markers to predict the genetic merit of candidate lines through genomic estimated breeding values (GEBVs) [7,9,10]. Earlier rice studies mainly examined GS accuracy for individual traits, such as grain yield, flowering time, grain quality, blast resistance, drought tolerance, and salinity tolerance, within one breeding population or a limited set of testing environments [10,11]. These studies compared genomic best linear unbiased prediction (GBLUP), Bayesian regression, kernel-based models, and other statistical approaches, with particular attention to marker density, training-population size, trait heritability, and genetic relatedness between training and prediction populations [12]. Prediction performance often declines when candidate lines are genetically distant from the training population or when genotype-by-environment interactions (G × E) alter trait expression across locations and seasons. Recent studies have expanded GS by incorporating high-throughput genotyping, high-throughput phenotyping (HTP), machine learning, and speed breeding, although these components are often discussed as separate technical developments. A more complete breeding framework should examine how genome-wide markers, image-derived phenotypic traits, environmental data, and rapid generation advancement can inform selection decisions within a single rice improvement pipeline.

Accordingly, this narrative review examines recent advances in rice genomic selection and their integration with high-throughput genotyping and HTP, machine learning, multi-environment prediction, and speed-breeding approaches. The review discusses the contribution of these technologies to the improvement of prediction accuracy, selection efficiency, and breeding-cycle duration, while identifying current limitations and priorities for future research. Relevant literature was identified through searches of the Web of Science Core Collection, Scopus, PubMed, and Google Scholar, with primary emphasis on peer-reviewed studies published between January 2015 and June 2026. Earlier seminal publications were included when they provided essential conceptual or methodological foundations. Studies were selected according to their direct relevance to the scope of the review, while duplicate records and publications with limited relevance to the review objectives were excluded.

## 2. Genomic Selection Platforms for Rice Improvement

GS predicts the genetic merit of a rice genotype from genome-wide marker data and typically estimates additive breeding values for selection [10,11]. Briefly, the procedure starts with a training population for which genome-wide genotype data and reliable phenotypic records are available, whereas candidate lines require only genotyping before they are ranked for selection [13]. Phenotypic data are commonly adjusted for location, year, replication, block effects, and management conditions through best linear unbiased estimates or best linear unbiased predictions before model development. In GBLUP, realized genetic similarity among lines is represented by a genomic relationship matrix calculated from marker data, and the model estimates the breeding value of each genotype from this matrix [14]. Ridge regression best linear unbiased prediction estimates small effects for all markers under a common variance assumption, whereas Bayesian models allow marker effects to differ in magnitude, and reproducing kernel Hilbert space (RKHS) models can capture non-additive genetic effects [14,15]. A study of 417 *indica* rice accessions genotyped with 24,502 single-nucleotide polymorphisms (SNPs) through Hyper-seq evaluated amylose content and gel consistency through five-fold cross-validation repeated 30 times [16]. Model performance was calculated as the Pearson correlation between GEBVs and reference values in the validation set. Under this correlation-based metric, GBLUP produced values of 0.8360 for amylose content and 0.7185 for gel consistency [16]. When SNPs associated with the target traits at *p*-value ≤ 0.1 were used, the reported correlations increased to 0.9669 for amylose content with 2431 markers and 0.9520 for gel consistency with 2453 markers [16]. These values represent unadjusted correlation-based performance and should not be interpreted as correlations with unobserved true breeding values or as heritability-adjusted prediction accuracy. Another study evaluated 459 rice varieties with 3955 SNPs for nine agronomic traits through five-fold cross-validation repeated nine times [17]. It reported correlation-based predictive performance ranging from 0.4066 to 0.8960 across traits and models. Mean values were 0.6629 for the combined-environment dataset, 0.5684 for Hangzhou (China), and 0.5391 for Lingshui (China) [17]. These averages summarize different traits and model combinations within that study and are not directly comparable with the trait-specific values reported as recently described [16]. More generally, comparisons among genomic prediction studies require consideration of the metric definition, phenotype used as the reference, cross-validation design, trait heritability, population structure, training-set composition, and relationship between training and validation materials [12,18,19].

The accuracy of GEBVs depends on marker quality, density, genomic distribution, and the genetic connection between the training and candidate populations, which makes platform selection and training-population design central components of GS [19,20,21,22]. Rice breeding programs can generate marker data through SNP arrays [16,17,21,23,24], genotyping-by-sequencing [22,25], low-coverage whole-genome sequencing [26], whole-genome resequencing [27], targeted amplicon sequencing, Kompetitive allele-specific PCR assays [28], and reduced-representation sequencing platforms (Table 1). Briefly, SNP arrays provide standardized genotypes at predefined loci for routine screening, whereas sequence-based platforms identify polymorphisms within the target germplasm and permit adjustment of marker density according to breeding objectives and available resources [16,17,21,23]. Targeted platforms are useful for quality control, varietal fingerprinting, and selection at validated major genes, whereas broad genome-wide coverage is generally needed for genomic prediction of complex traits controlled by numerous loci with small effects. For example, Spindel et al. (2015) genotyped 363 elite tropical rice breeding lines with 73,147 genotyping-by-sequencing markers and reported that a density of approximately one marker per 0.2 cM was sufficient to maintain prediction performance within the evaluated population [29]. Arbelaez et al. developed the 1k-RiCA panel with approximately 1000 SNPs distributed across the rice genome, including genome-wide, trait-specific, and quality-control markers for genomic prediction and breeding applications in elite *indica* germplasm [24]. Training populations should include elite parents, advanced lines, and representative progeny from current crossing cycles, with phenotypic records collected across the target environments [13,20]. For example, Bartholomé et al. (2023) used 241 reference accessions genotyped with 20,255 SNPs to predict performance in a breeding population of 393 lines, with shared parental backgrounds linking the reference and breeding materials [30]. Such a population design can strengthen the genetic connection between training lines and future candidates and may improve prediction when subsequent breeding cycles remain related to the training material [19].

After marker data and training populations have been established, the statistical model determines how genome-wide marker information is converted into GEBVs [11,29]. GBLUP estimates additive genetic values from a genomic relationship matrix, whereas ridge regression best linear unbiased prediction estimates effects across all markers under a common marker-variance assumption [14,15]. These models provide efficient baseline approaches for polygenic traits such as grain yield, flowering time, plant height, and panicle architecture (Table 2). Bayesian models, including BayesA, BayesB, BayesC, BayesR, and Bayesian least absolute shrinkage and selection operator, permit unequal marker-effect variances and may assign greater importance to loci linked with large-effect quantitative trait loci [31,32]. Meanwhile, RKHS models use marker-derived kernel matrices to capture nonlinear genetic effects and potential epistatic interactions [32]. Model performance should be evaluated through cross-validation schemes that represent the intended selection scenario in a breeding program (Table 2). For example, Bhandari et al. (2019) compared GBLUP with RKHS regression models for drought-related rice traits and reported improved predictive ability from trait-specific markers and multi-environment prediction models [33], while Ben Hassen et al. (2018) evaluated GBLUP and RKHS approaches under alternate wetting and drying and continuous flooding conditions, where models that incorporated multiple environments improved prediction for breeding lines tested across contrasting water regimes [34]. Muvunyi et al. (2022) reported higher predictive ability for grain trace-element concentrations from multi-trait models when correlated auxiliary traits were available [35]. In contrast, Ahmadi et al. (2021) assessed grain arsenic concentration and grain yield across environments and found that multi-environment and multi-trait multi-environment models improved prediction relative to single-environment analyses [36]. Within the populations and validation schemes examined, these studies suggest that GBLUP and ridge regression best linear unbiased prediction can serve as baseline models, while Bayesian, RKHS, multi-trait, and multi-environment approaches may be advantageous for traits influenced by major loci, correlated phenotypes, nonlinear effects, or G × E [9,10,12,31,32].

GS has been evaluated for rice breeding targets related to productivity, grain quality, and disease resistance, although the reported prediction metrics and validation designs differ among studies (Table 3). For grain yield, genomic models can rank breeding lines for yield potential, flowering time, plant height, and related yield components before extensive multi-environment evaluation [10,29]. Particularly, Nguyen et al. (2023) evaluated 111 elite irrigated rice lines across 15 environments and defined predictive ability as the Pearson correlation between predicted values and adjusted phenotypes in the validation environment. Under three validation scenarios for untested environments, the reported correlations for grain yield ranged from −0.29 to 0.62 [19]. These results indicate strong variation among environments but should not be directly compared with reliability-adjusted accuracies or correlations obtained through random within-population cross-validation. Grain-quality studies have examined amylose content, alkali spreading value, grain dimensions, chalkiness, milling recovery, and eating quality [10,29]. Yu et al. (2022) compared several genomic prediction algorithms for yield and grain-quality traits, but the reported performance must be interpreted according to the study-specific cross-validation procedure and correlation metric [37]. Furthermore, disease-resistance studies have similarly used different population structures, pathogen isolates, marker-weighting procedures, and validation schemes. Akohoue et al. (2026) evaluated leaf blast and panicle blast resistance in two rice populations and reported that GBLUP models weighted with genome-wide association study-derived marker information increased predictive ability by 4–37% relative to conventional GBLUP models within the evaluated datasets [38]. These percentage gains describe within-study model contrasts and should not be interpreted as absolute prediction accuracies or directly compared with correlation coefficients from other populations.

Applications under abiotic stress are particularly relevant for smart and greener agriculture (Table 4). In a rainfed lowland rice panel of 280 accessions genotyped with 215,000 SNPs, Bhandari et al. (2019) reported increases in predictive ability of up to 22% from trait-specific marker sets and up to 32% from multi-environment models under the validation scenarios examined [33]. Salinity-tolerance prediction can target shoot biomass, root traits, tiller number, sodium concentration, potassium concentration, and stress-response indices under control and salt-stress conditions. Bartholomé et al. (2023) used 20,255 SNPs from 241 *japonica* accessions to train models for eight morphological traits and ion mass fractions, then predicted the performance of 393 breeding lines under mild salinity stress [30]. Nutrient-use efficiency, particularly nitrogen-use efficiency, represents another important application in low-input rice systems. Genomic prediction can support selection for grain yield under low nitrogen supply, nitrogen uptake, nitrogen utilization, biomass accumulation, and yield stability across nitrogen regimes. Rakotomalala et al. (2021) reported much lower predictive ability when a diversity panel was used to predict a genetically distinct upland rice breeding population, which suggests that training populations may require regular updating with materials from current breeding cycles [43]. These examples suggest that GS design should account for trait genetic architecture, target production environments, measurement protocols, and genetic relatedness between training and candidate populations.

Population structure and genetic relatedness can inflate genomic prediction performance when close relatives, progeny from the same parents, or members of the same genetic subgroup occur in both training and validation sets, since random k-fold cross-validation may capture shared ancestry, subgroup-specific allele frequencies, and common linkage disequilibrium patterns that may not persist in future breeding materials [29,45,46]. Consequently, random *k*-fold cross-validation is most informative for prediction within a genetically connected population, whereas leave-one-family-out validation evaluates transferability across parental combinations, forward-cycle validation assesses prediction in later breeding cycles, cross-year validation examines performance in future seasons, cross-environment validation evaluates transferability to untested locations or management conditions, and independent external validation tests materials that were not used during model development. The importance of validation design is evident from rice studies in which prediction performance changed sharply across populations and environments. For example, Nguyen et al. (2023) reported grain-yield predictive abilities from −0.29 to 0.62 across 15 environments [19], while Zhang et al. (2023) obtained a mean predictive ability of 0.6629 for a multi-environment model compared with 0.5684 and 0.5391 for separate analyses at Hangzhou and Lingshui (China) [17]. Bartholomé et al. (2023) reported cross-validation values from 0.25 to 0.64 for salinity-related traits and an independent validation value of 0.66 for selected traits [30], whereas Rakotomalala et al. (2021) observed across-population predictive abilities of only 0.06–0.27 when a diversity panel was used to predict a genetically distinct breeding population [43]. Across the studies examined, high performance under random partitioning may not translate into reliable prediction of new families, future cycles, unrelated germplasm, future years, or new environments, so GS studies in rice should report the genetic relationship between training and validation materials, environmental overlap, population partitioning, and at least one validation design that corresponds to the intended breeding application.

Validation schemes should be selected according to the breeding scenario that the genomic model is intended to support. Leave-one-family-out validation withholds all progeny from one family and evaluates whether information from other families can predict the excluded family, which provides a more demanding assessment of prediction across parental combinations. Forward prediction across breeding cycles uses earlier cycles for model training and a subsequent cycle for validation, thereby testing the effects of changing parental contributions, allele frequencies, linkage phases, and selection history [45]. G × E creates an additional limitation when the rank of rice genotypes changes across locations, seasons, water regimes, soil types, fertilizer levels, or stress intensities [45,46]. Zhang et al. (2023) evaluated nine agronomic traits in two rice-growing environments and reported a mean predictive ability of 0.6629 for the multi-environment model, compared with 0.5684 and 0.5391 for separate single-environment analyses [17]. Bhandari et al. (2019) reported higher predictive ability from multi-environment models and trait-specific marker sets for drought-related traits, which supports the inclusion of representative target environments in the training dataset [33]. Prediction transferability can further decline across breeding cycles as new parental combinations alter allele frequencies, linkage phases, and haplotype composition. Differences in phenotyping protocols, genotyping platforms, marker imputation quality, and field management may also reduce agreement between historical training datasets and new candidate populations [45,46]. Rice breeding programs should therefore update training populations after each cycle with elite parents and recent progeny, maintain phenotypic records across representative target environments, and evaluate models through independent validation sets that represent future selection candidates instead of random cross-validation alone [7,19,25].

## 3. High-Throughput Phenotyping and Machine Learning for Genomic Prediction

**Figure 1:**
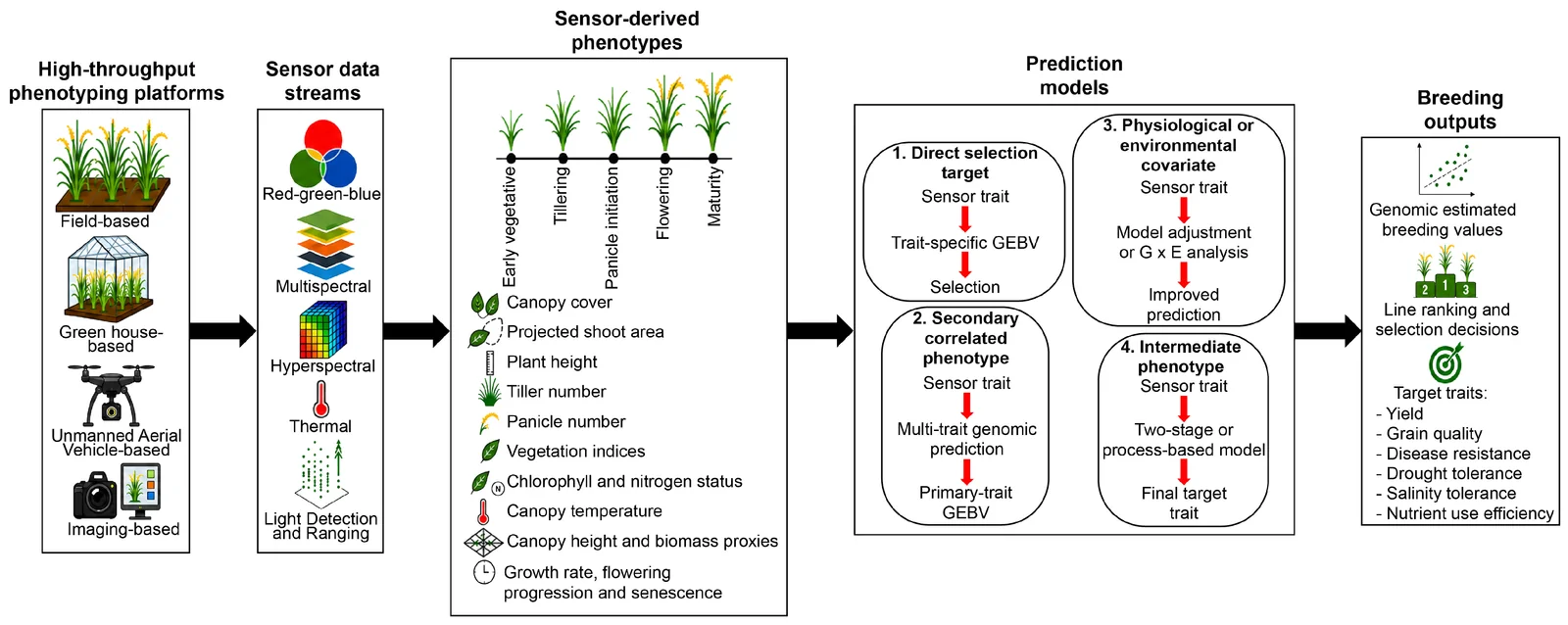
Alternative pathways for integrating high-throughput phenotypes into rice genomic prediction. Sensor-derived traits may serve as direct selection targets, secondary correlated phenotypes in multi-trait models, physiological or environmental covariates, or intermediate phenotypes in two-stage and process-based models.

HTP provides repeated and objective measurements of rice growth, canopy development, stress response, and yield-related traits at scales beyond practical manual assessment [47,48,49,50]. Field-based HTP systems use handheld sensors, fixed cameras, ground vehicles, gantries, or mobile sensor platforms to quantify canopy height, tiller density, canopy cover, biomass, flowering date, leaf angle, lodging, and spectral vegetation indices under target production conditions [51]. Greenhouse-based systems support controlled evaluation of rice materials under defined water, salinity, temperature, and nutrient treatments through automated imaging chambers or conveyor-based platforms. For example, Kim et al. (2020) developed a rice drought-phenotyping platform that integrated red-green-blue (RGB), near-infrared, infrared, and chlorophyll-fluorescence imaging with gravimetric measurements of water use efficiency and transpiration rate [52]. RGB images produced traits such as projected plant area, greenness, compactness, convex-hull area, and plant width, whereas near-infrared images estimated plant water status, thermal images measured plant temperature, and chlorophyll fluorescence assessed photosynthetic performance [51,53]. Unmanned aerial vehicle (UAV)-based phenotyping extends field assessment from individual plots to large breeding nurseries through RGB, multispectral, hyperspectral, thermal, and Light Detection and Ranging (LiDAR) sensors [54,55]. Jiang et al. (2021) used UAV-RGB imagery, digital orthophotography maps, and canopy-height models to characterize the temporal drought response of rice germplasm under field conditions [56]. Wang et al. (2021) used UAV-based hyperspectral imagery and lodging information to classify yield performance among *japonica* rice lines [57], whereas Bai et al. (2023) applied time-series UAV data and environmental information to assess bacterial leaf blight severity and identify resistance-associated genomic regions [58]. Imaging-based phenotyping provides morphological, spectral, physiological, and disease-related traits that can serve as direct selection criteria or auxiliary variables in genomic prediction models for grain yield, drought adaptation, nitrogen status, and disease resistance (Figure 1) [48].

The contribution of each sensor modality differs according to the structural, physiological, or stress-related trait under assessment [59,60]. RGB images provide projected plant area, canopy cover, green-pixel fraction, color indices, tiller angle, tiller number, canopy height, and lodging-related traits. For example, Wu et al. (2018) extracted 739 dynamic traits from micro-computed tomography and RGB images of 234 rice accessions at nine tillering stages, and two early tiller traits accounted for 30% of the observed grain-yield variation within the evaluated dataset [61]. Time-series RGB images from UAVs can also produce digital surface models for canopy-height analysis [55]. Taniguchi et al. (2022) reported that maximum canopy height contributed to the prediction of culm length in 30 Japanese rice cultivars evaluated across three years, while canopy-height trajectories supported the prediction of heading date and biomass within the same dataset [62]. Multispectral sensors record visible, red-edge, and near-infrared bands that support the calculation of vegetation, chlorophyll, and water-sensitive indices [60,63]. These variables can quantify canopy vigor, leaf area index, biomass accumulation, nitrogen status, senescence progression, and water limitation. Recently, five-band reflectance data at 490, 550, 670, 720, and 850 nm were used to estimate rice leaf area index across tillering, jointing, booting, and heading stages [63]. Hyperspectral imaging provides much narrower and more continuous spectral information than multispectral systems, which permits characterization of chlorophyll concentration, leaf nitrogen content, pigment composition, leaf water status, and disease-associated spectral responses. For example, Maina et al. (2023) analyzed 400–1000 nm leaf spectra from three rice genotypes exposed to three nitrogen levels and three *M. oryzae* isolates, which enabled differentiation of healthy tissue and blast symptom classes under contrasting nitrogen supply [64]. Thermal imaging measures canopy or leaf temperature and provides traits related to transpiration, evaporative cooling, water-use efficiency, and drought response [59]. Kim et al. (2020) combined RGB, near-infrared, thermal infrared, chlorophyll-fluorescence, and gravimetric measurements in a rice drought platform, where RGB traits quantified plant area and compactness, near-infrared images estimated water content, thermal images assessed plant temperature, and fluorescence measured photosynthetic efficiency [52]. LiDAR systems generate three-dimensional point clouds that support non-destructive estimation of canopy structural traits, including canopy height, canopy volume, and height distribution. LiDAR-derived canopy height and density have been used for non-destructive estimation of rice canopy structure and leaf area index. These repeated sensor-derived traits can serve as direct selection targets or auxiliary phenotypes for genomic prediction models that target grain yield, drought adaptation, nutrient-use efficiency, and disease resistance.

Sensor-derived phenotypes can contribute to rice genomic prediction through four statistically and biologically distinct pathways. First, canopy height, canopy temperature, biomass, water use, spectral indices, or disease severity can serve as direct selection targets when the measured trait has agronomic value, sufficient heritability, repeatable measurement, and genetic variation that supports selection. Second, a sensor-derived trait can serve as a secondary phenotype in a multi-trait genomic model for a primary breeding target such as grain yield, where improvement depends on the genetic correlation between traits, the heritability and measurement precision of the secondary trait, and its availability in the training population or candidate lines. Under a prediction of untested genotypes design, secondary phenotypes are available only for the training population and contribute through genetic relationships, whereas a prediction of genotypes evaluated in at least one environment design also includes secondary phenotypes from candidate lines and can produce greater gains through direct information on those candidates. In 357 rice accessions assessed daily for projected shoot area and water use over 20 days, multi-trait random regression models exceeded single-trait models, with the greatest improvement when projected shoot-area records were available for the validation accessions [65]. Third, physiological measurements such as canopy temperature or water-status indices can enter a model as covariates that account for stress exposure, developmental differences, or environmental variation without serving as selection targets. Fourth, growth rate, leaf area, tiller development, or other physiological characteristics can act as intermediate phenotypes within a two-stage or process-based model that links genomic and environmental information with a final target trait. In a two-year rice experiment, a model that first predicted growth-related intermediate traits and then used these predictions with environmental data produced biomass correlations of 0.53 and 0.65, compared with 0.40 and 0.65 from direct genomic prediction in the respective years [66]. HTP-derived traits are most likely to improve genomic prediction when they are heritable, genetically associated with the primary trait, measured at an informative developmental stage, less expensive or earlier to obtain than the primary trait, and available under conditions that correspond to the intended selection scenario. Limited genetic correlation, strong environmental sensitivity, unsuitable measurement timing, redundant predictor information, or secondary-trait measurements that are unavailable for candidate lines can reduce or remove the expected advantage.

Beyond single-time-point measurements, repeated RGB imaging can generate daily projected shoot area, canopy cover, tiller expansion rate, plant compactness, and growth trajectories, while multispectral, thermal, and LiDAR measurements can provide temporal trajectories for vegetation indices, canopy temperature, canopy height, biomass accumulation, and water-use responses. These data can be incorporated into genomic prediction through multi-trait models that treat measurements from different dates as correlated phenotypes, or through random regression models that estimate genotype-specific trait trajectories across time. Campbell et al. (2018) used random regression models with Legendre polynomials to predict projected shoot-area trajectories in a rice diversity panel genotyped with 33,674 SNPs, and the longitudinal approach captured more genetic variation than separate analyses at individual time points [65]. Baba et al. (2020) extended this framework in 357 rice accessions phenotyped daily for projected shoot area and water use over 20 days under a greenhouse HTP system [67]. Their multi-trait random regression model used the genetic association between biomass development and water use to improve the prediction of daily water-use trajectories. Temporal datasets can also be summarized as maximum values, growth rates, inflection points, cumulative area under the curve, or functional principal component scores before inclusion in genomic models. Such temporal features can identify lines with rapid early biomass accumulation, stable canopy cooling during water deficit, delayed senescence, or efficient water use before final grain yield is available, which supports earlier selection in rice breeding programs.

Machine learning and deep learning models do not provide an automatic advantage over GBLUP, Bayesian regression, or RKHS, since their performance depends on population size, marker dimensionality, trait architecture, genetic relatedness, feature selection, hyperparameter optimization, and validation design. A benchmark across 14 plant-breeding datasets, which contained 318–1403 genotypes and 2038–78,606 markers and included *indica* and *japonica* rice populations, showed that neither deep learning nor GBLUP achieved superior performance across all traits, with GBLUP remaining competitive for traits dominated by additive effects and deep learning providing advantages for several complex traits and ranking tasks [68]. In a separate analysis of 322 maize (*Zea mays*) lines and 646 eucalyptus progenies, a long short-term memory model produced predictive abilities from 0.27 to 0.78 and exceeded GBLUP, RKHS, and Bayesian models for the evaluated traits, although the comparison used random 90:10 cross-validation and extensive architecture tuning, which limits conclusions about transfer to unrelated families or future breeding cycles [69]. Deep neural network genomic prediction achieved performance comparable with conventional models in small datasets and higher accuracy in the evaluated large multi-omics datasets, with computation times up to tenfold lower than DeepGS. These findings suggest that deep learning may offer advantages when sufficiently large datasets and heterogeneous inputs are available. In smaller marker-only rice populations, simpler models may provide similar or better out-of-sample performance with lower computational demand and greater model transparency, so machine learning and deep learning should be compared with GBLUP, Bayesian regression, and RKHS under the same population partitions, phenotype adjustments, and validation metrics, with imputation, feature selection, scaling, and hyperparameter optimization conducted within each training fold to prevent data leakage.

Data leakage can inflate genomic and phenomic prediction performance when information from validation observations contributes to model development, data preprocessing, or predictor construction. This risk is particularly high in longitudinal HTP datasets when measurements from the same genotype, plot, family, year, environment, or temporal trajectory occur in both training and test sets, since the model may recognize genotype-specific growth patterns or shared environmental signals instead of associations that transfer to new breeding materials. Consequently, data partitions should be created at the level of the intended prediction unit, with all repeated observations from one genotype, family, plot, year, or environment assigned to the same partition. Imputation, normalization, environmental correction, feature selection, dimensionality reduction, temporal feature extraction, and sensor calibration must be fitted within each training partition and then applied to the corresponding validation partition. A machine-learning assessment outside plant breeding reported increases in Pearson correlation of 0.03–0.47 after feature selection before data partitioning and 0.04–0.28 after the inclusion of repeated subjects, which illustrates the potential inflation associated with shared observations and preprocessing information [70,71]. Plant prediction studies have addressed this problem through feature selection within each cross-validation fold and through nested cross-validation in which an inner loop selects features and hyperparameters while an independent outer loop estimates model performance [71,72]. For multi-environment and temporal rice datasets, validation should group observations by genotype, family, year, site, or complete growth trajectory according to the intended breeding application, while hyperparameter optimization and model selection should use only the inner training folds, since repeated access to the outer validation set converts model evaluation into an additional stage of model development.

## 4. Speed Breeding and Integrated Breeding Pipelines for Accelerated Genetic Gain

Speed breeding in rice reduces the seed-to-seed interval through stage-specific manipulation of photoperiod, light spectrum, light intensity, temperature, humidity, plant density, nutrient supply, and premature seed recovery [50,73]. Its biological basis lies in the separation of the vegetative period into a basic vegetative phase, during which young plants show limited photoperiod sensitivity, and a subsequent photoperiod-sensitive phase, during which short days promote panicle initiation [74]. For example, the SpeedFlower protocol was designed for fully enclosed and programmable growth chambers equipped with tunable light-emitting diode channels, automated irrigation, climate-control software, controlled air circulation, humidity regulation, and carbon dioxide delivery. It applies a red-dominant spectrum at 800 µmol m^−2^ s^−1^, a 24-h photoperiod during the first 15 days after sowing, and a subsequent 10-h photoperiod until anthesis and seed harvest [75]. The system maintains 32/30 °C day/night temperatures and 65% relative humidity, with supplementary far-red treatments after the initial vegetative phase. Panicles are harvested 13–15 days after anthesis, dried at 38 °C, and treated with gibberellic acid to promote rapid germination. This protocol completed one generation within 58–71 days across a validation panel of 198 accessions from all 12 *O. sativa* population groups, which corresponded to approximately 5.1–6.3 generations per year [75]. In contrast, the SpeedyPaddy protocol provides a lower-cost operational alternative for large breeding populations through the use of a fiber-sheet screenhouse, portable benches, plug trays, halogen-based lighting, heaters, and a humidifier [76]. It uses dense planting at approximately 700 plants m^−2^ to suppress tillering and reduce plant height, with 13 h light and 11 h dark during seedling and vegetative development, followed by 8 h light and 16 h dark during reproductive development [76]. Light intensity is maintained near 750–800 µmol m^−2^ s^−1^, with day and night temperatures of 30–32 °C and 23–25 °C, respectively, and relative humidity near 70% [76]. Nutrient inputs are supplied through limited fertigation to maintain compact growth, while seeds harvested 15 days after anthesis are dried at 38 °C and treated with 100 ppm gibberellic acid or 2% calcium chloride before direct sowing [76]. SpeedyPaddy achieved one generation within 68–75 days, supported four to five generations per year, accommodated about 15,680 plants per cycle, and reduced the breeding cycle by 51, 60, and 76 days in early-, medium-, and late-duration rice varieties, respectively [76]. Thus, SpeedFlower is suited to highly controlled facilities that prioritize environmental precision and broad germplasm validation, whereas SpeedyPaddy provides a scalable system for programs that require high population throughput under limited infrastructure and operating budgets [75,76].

The controlled systems described above provide the short generation intervals required to convert genomic predictions into repeated selection cycles within a breeding program. After crossing, large F_2_ or F_3_ families can be established under SpeedFlower or SpeedyPaddy conditions [75,76], genotyped at the seedling stage, and ranked with genomic estimated breeding values for traits that are costly or unreliable to assess in early generations, such as grain yield, drought adaptation, grain quality, and nutrient-use efficiency. Only a selected proportion of high-ranking plants is then advanced through dense planting, single-seed descent, or controlled selfing, while the remaining plants are discarded before they consume space in subsequent cycles [77]. This approach combines speed breeding for rapid generation turnover with GS for early ranking among thousands of related individuals before full phenotypic evaluation. For example, a breeding population derived from two elite parents can be genotyped in the F_2_ generation, screened for favourable alleles at major loci through marker-assisted selection, ranked by genomic estimated breeding values for yield and flowering time, and advanced under rapid cycling until F_5_ or F_6_. Near-fixed lines can then enter multi-location trials, whereas the top early-generation lines can also be recycled immediately as parents for the next crossing cycle. In hybrid rice, genomic prediction can further rank potential male and female parental combinations before large-scale hybrid seed production, which reduces the number of crosses required for field testing [39]. Rapid cycling is also valuable for recurrent GS, where selected plants are crossed after each prediction round to increase the frequency of favorable alleles across cycles. The value of this integration is illustrated by SpeedFlower, which was designed for rapid crossing, inbreeding, mapping-population development, GS, and genome editing [75], and by SpeedyPaddy, which supported the advancement of segregating generations, hybridization, marker-assisted introgression, trait phenotyping, and quantitative trait locus mapping [76]. Thus, GS directs which plants and crosses should move forward, while rapid cycling determines how quickly those decisions generate the next breeding population.

**Figure 2:**
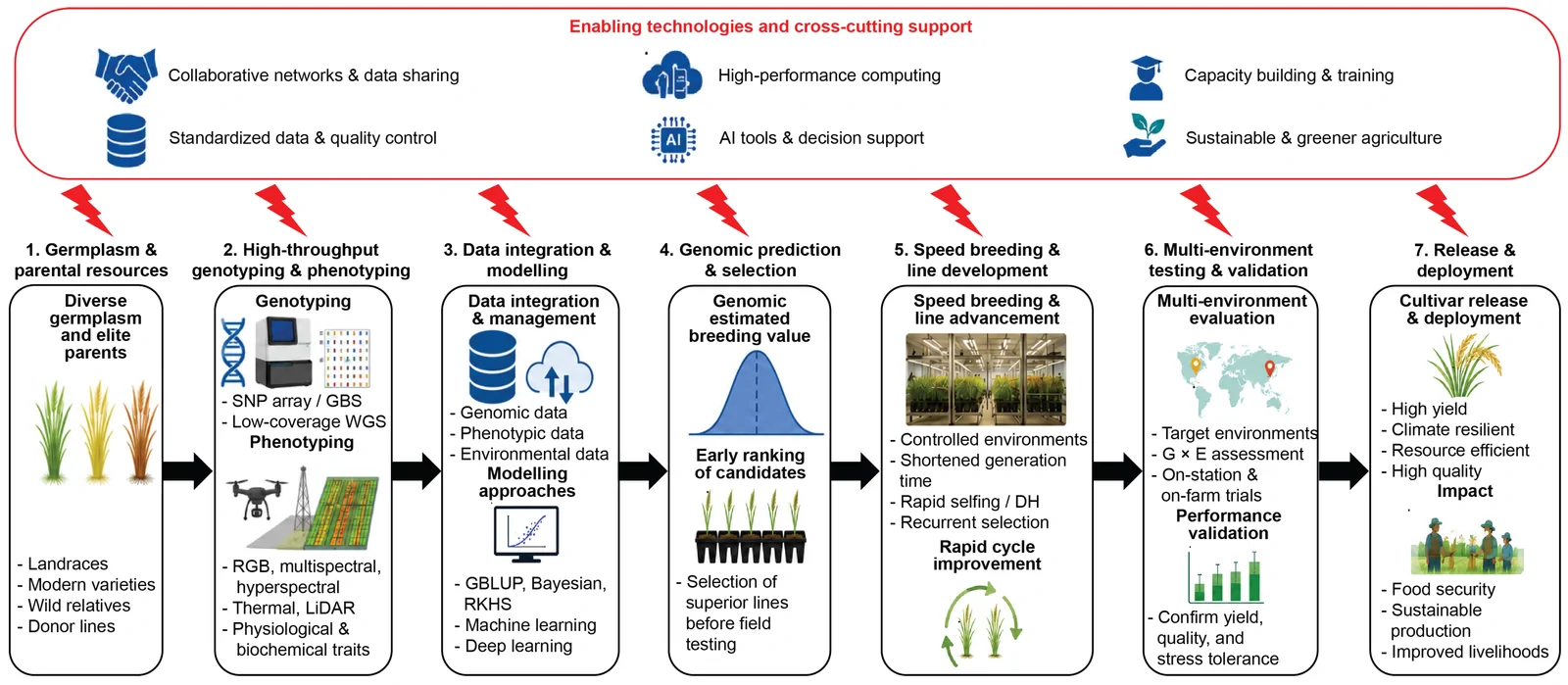
Integrated pipeline for genomic selection combined with high-throughput phenotyping and speed breeding in rice improvement. GBS, genotyping-by-sequencing; WGS, whole-genome sequencing; HTP, high-throughput phenotyping; GEBV, genomic estimated breeding value; DH, doubled haploid; G × E, genotype-by-environment interaction.

Building on early-generation ranking and rapid cycling, an integrated rice breeding pipeline should link crossing design, genome-wide genotyping, HTP, genomic prediction, and controlled generation advancement within a continuous data cycle [50]. Crosses are first selected from parental genomic estimated breeding values, complementary haplotypes, and target trait combinations, after which large F_2_ or F_3_ populations are genotyped and screened for major alleles and genome-wide breeding values (Figure 2). Speed-breeding facilities then advance selected plants rapidly to later inbreeding generations, while greenhouse and field phenotyping collect repeated measurements of canopy growth, flowering time, plant height, spectral indices, stress responses, and grain traits from a representative subset [50,73,74]. These phenotypic records are added to the training population after quality control and used to update genomic prediction models for the next cycle, which helps maintain prediction accuracy as parental combinations and allele frequencies change. Multi-environment field trials remain necessary for advanced lines, particularly for grain yield and stress adaptation, but their main role shifts from screening all breeding materials to validating a smaller group of genomically selected candidates across target production environments [47,48]. For example, the evaluation of 111 elite lines across 15 environments provides a case study of how historical multilocation data may inform operational selection models within genetically connected breeding materials [19]. In another upland rice program, multi-generation and multi-location information improved recurrent GS under the evaluated conditions, which offers a case study for the integration of successive selection cycles and field evaluation [45]. HTP can provide auxiliary information for this pipeline. The value of combining phenomic and genomic data should be evaluated across traits, populations, and target environments through independent validation [47,52]. Thus, integration should be designed as an iterative breeding architecture in which genomic prediction guides early selection, phenotyping updates prediction models, validates physiological responses, and speed breeding compresses the time between one selection decision and the next.

Although this iterative breeding architecture can shorten the interval between successive selection decisions and increase medium-term genetic gain, its long-term value also depends on the preservation of additive genetic variance across recurrent cycles. Genomic prediction and speed breeding can support larger candidate populations, stronger selection intensity, and faster recycling of superior parents, but repeated use of a small group of high-GEBV lines can reduce effective population size, increase genomic coancestry and inbreeding, and narrow the genetic base available for future improvement. Simulation analyses in rice indicated that a two-year genomic-selection cycle increased medium-term genetic gain by 22–27% compared with a three-year cycle under different levels of G × E [19], although this advantage may decline after repeated cycles when favorable alleles approach fixation and additive genetic variance decreases. A long-term soybean (*Glycine max*) simulation further showed that across-family and pre-selected-family strategies depleted 80% of the initial genetic variance within 28–58 cycles, while within-family selection retained genetic variance for up to 184 cycles and generated higher long-term gain [78]. Consequently, rice breeding programs should balance the rapid advancement of high-value lines with controlled parental contributions, minimum family representation, avoidance of repeated crosses among closely related parents, and selection of crosses based on both expected progeny performance and within-cross variation. In a wheat (*Triticum aestivum*) breeding population, cross-selection criteria that incorporated progeny variance increased the genetic value of superior progeny by up to 5%, improved genetic gain by up to 4%, and retained up to 4% more genic variance than criteria based only on parental mean [79]. Optimum contribution selection, genomic mate allocation, periodic introduction of diverse donor material, and routine monitoring of genomic inbreeding, pairwise coancestry, effective population size, allele-frequency change, and additive genetic variance can support rapid short-term progress without exhausting the genetic resources required for later breeding cycles.

## 5. Future Directions

Future priorities for rice improvement should be evaluated according to the maturity of each technology and the strength of evidence supporting its use in breeding programs. Genome-wide prediction, multi-environment evaluation, high-throughput phenotyping, rapid generation advancement, and digital breeding-data management have reached operational or near-operational application in several rice programs. The OneRice Breeding Framework, for example, provides an end-to-end structure that links product profiles, parental selection, breeding pipelines, testing networks, and cultivar deployment, although the level of implementation may differ among national and regional programs [80]. These established components can be incorporated into current breeding pipelines, provided that training populations, phenotyping procedures, environmental characterization, data quality, and independent validation meet the requirements of the target breeding program.

Multi-omics prediction remains at an experimental stage in plant breeding and has not reached routine application in rice selection pipelines. Genomic, transcriptomic, metabolomic, proteomic, and epigenomic datasets can provide complementary information on complex traits, but their value depends on tissue type, developmental stage, environmental treatment, population size, data-processing method, and prediction design. A study in *Arabidopsis thaliana* reported that transcriptome- and methylome-based models achieved performance comparable to genome-based models for several complex traits, although additional molecular layers did not provide a uniform advantage across all traits [81]. Rice transcriptomic and metabolomic studies have identified molecular processes associated with heterosis, grain quality, stress response, and nutrient composition [80], but most have focused on biological discovery and have not tested prospective selection across independent breeding cycles. Multi-omics integration currently has experimental support, while operational adoption will depend on lower assay costs, standardized sampling, larger training populations, and prospective evidence of prediction gains beyond genomic and phenotypic information.

Crop simulation models occupy an intermediate position between experimental demonstration and breeding application. Rice studies that combined genomic prediction with growth-related intermediate traits and environmental information provide preliminary evidence that process-based models may support prediction of biomass, flowering, canopy development, and stress response under defined experimental conditions. Wider use will require calibration across genotypes, years, management systems, and target environments, followed by validation in breeding populations that were not used for model development. Automated decision-support tools for data-quality control, cross prioritization, trial allocation, and candidate ranking remain mainly at the proof-of-concept or early implementation stage, while a digital twin that continuously simulates the genetic composition, flowering behavior, stress response, and field performance of candidate rice crosses remains prospective. Current agricultural digital-twin studies mainly address crop monitoring, resource management, controlled environments, and production decisions, and they do not provide sufficient evidence for an operational rice breeding twin with validated cross-to-cultivar prediction [82]. Such a system would require integrated pedigree, genomic, phenomic, environmental, crop-model, and seed-inventory data, as well as prospective validation of prediction uncertainty across breeding cycles and locations. Digital twins capable of evaluating candidate crosses should therefore be presented as a long-term research direction and not as a mature component of current rice breeding pipelines.

Low-cost breeding platforms are essential for rice-growing regions where advanced controlled-environment facilities, dense marker panels, UAVs, hyperspectral sensors, and high-performance computing are unavailable [83]. Practical systems can combine low-density SNP panels, targeted Kompetitive Allele Specific PCR assays, smartphone-based imaging, inexpensive RGB cameras, handheld canopy sensors, open-source phenotyping software, shared genotyping services, and semi-controlled rapid-generation facilities. Public breeding programs could establish regional hubs that provide centralized genotyping, cloud-based data processing, and standardized phenotyping support for smaller national programs [84]. Breeding decisions should use tiered selection, with early generations assessed through low-cost markers and simple phenotypic measures, followed by more intensive genomic and multi-environment evaluation only for top-ranked lines. This structure would allow programs to maintain large population sizes and shorten breeding cycles without requiring every station to purchase expensive equipment.

Finally, data sharing and breeder capacity development are likely to influence the adoption of integrated rice breeding systems beyond a small number of well-resourced institutions [4,50]. Programs should use shared trait ontologies, standardized field books, unique identifiers for germplasm and seed lots, machine-readable metadata, version-controlled analysis pipelines, and clear records of genotyping, phenotyping, environmental conditions, and model updates. Data governance frameworks should define ownership, access rights, benefit sharing, licensing, privacy safeguards, and procedures for the responsible use of farmer, breeder, and institutional data. Findable, Accessible, Interoperable, and Reusable principles emphasize metadata and standards that support data discovery, access, interoperability, and reuse, while assessment tools can identify missing metadata and compliance gaps before datasets are incorporated into collaborative breeding networks. Capacity development should include quantitative genetics, experimental design, data stewardship, field phenotyping, remote sensing, programming, genomic prediction, and interpretation of uncertainty in breeding decisions.

## 6. Conclusions

The central message of this review is that the greatest value of GS in rice will arise from its operation as the decision core of an integrated breeding system that connects genome-wide genotyping, sensor-based phenotyping, environmental characterization, and accelerated generation advancement. This integration can increase selection intensity, shorten breeding cycles, and direct field resources toward candidates with the highest predicted value, although genetic gain will remain limited when training populations are poorly connected to future breeding materials, phenotypic records are not comparable across sites and years, validation schemes do not represent operational selection scenarios, or recurrent use of a narrow parental base erodes additive genetic variation. Major challenges also include the cost of advanced phenotyping platforms, unequal access to computational infrastructure, fragmented data standards, limited breeder expertise in quantitative and digital methods, and insufficient prospective evidence for multi-omics models, crop simulation frameworks, and artificial-intelligence tools across independent breeding cycles. Future research should prioritize forward-cycle and cross-environment validation, regular renewal of training populations, trait-specific assessment of HTP, harmonized metadata and quality-control procedures, and selection strategies that balance short-term genetic gain with effective population size and long-term genetic diversity. Practical implementation should establish tiered and affordable pipelines that combine low-cost marker panels, RGB or smartphone phenotyping, shared analytical services, and scalable speed-breeding facilities, particularly for public programs in resource-limited rice-growing regions. Progress in these areas will determine whether integrated genomic breeding becomes a routine approach for delivering high-yielding, climate-resilient, resource-efficient, and high-quality rice cultivars under increasingly variable production conditions.

## Figures and Tables

**Table 1:** Overview of genotyping platforms for genome-wide and targeted marker-data generation in rice breeding programs. The platforms differ in genomic coverage, marker type, throughput, cost, and suitability for applications such as genomic selection, marker-assisted selection, germplasm characterization, parental selection, and quality control.

Platform	Marker Data	Main Strengths	Main Limitations	Principal Breeding Uses
SNP array	Predefined genome-wide SNPs	Reproducible, rapid, and low missing-data rate	Restricted to previously identified variants	Quality control, parental characterization, and GS
Genotyping-by-sequencing	Genome-wide SNPs from reduced-representation sequencing	Moderate cost and suitable for large populations	Variable read depth and frequent need for imputation	Diversity analysis, linkage mapping, and GS
Low-coverage whole-genome sequencing	Genome-wide SNPs obtained through shallow sequencing and imputation	Broad coverage and flexible marker density	Requires reference panels and bioinformatic support	Large-scale genomic prediction and haplotype analysis
Whole-genome resequencing	Dense SNPs, insertions, deletions, structural variants, and haplotypes	Comprehensive detection of genetic variation	High sequencing, storage, and computational costs	Allele discovery, founder characterization, and reference-panel construction
Targeted amplicon sequencing	SNPs and haplotypes in selected genes or genomic regions	High depth, low cost per locus, and easy multiplexing	Limited coverage for highly polygenic traits	Marker-assisted selection, allele tracking, and gene validation
Kompetitive allele-specific PCR assays	Individual SNP alleles at predefined loci	Rapid, inexpensive, and compatible with routine laboratories	Unsuitable for genome-wide prediction at low marker numbers	Selection for major resistance, stress-tolerance, and grain-quality genes
Hyper-seq	Reduced-representation genome-wide SNPs	High throughput, low cost per sample, and adjustable marker density	Requires sequencing, filtering, and imputation	GS, association analysis, and quality-trait prediction
DArTseq	Genome-wide SNPs and presence-absence markers	High multiplexing capacity across diverse germplasm	Marker coverage depends on platform design and commercial access	Diversity analysis, population structure, parental selection, and preliminary genomic prediction

**Table 2:** Comparison of major genomic prediction approaches based on model assumptions, strengths, limitations, computational demand, and breeding applications.

Method	Assumption	Strength	Limitation	Computing Demand	Suitable Use
GBLUP	Many small additive effects	Stable and simple	Weak for major or nonlinear effects	Low	Routine prediction of polygenic traits
Bayesian methods	Unequal marker effects	Captures major and minor loci	Sensitive to prior choice	Moderate-high	Quality, resistance, and oligogenic traits
RKHS	Nonlinear genetic relationships	Models epistasis and non-additive effects	Limited interpretability	Moderate-high	Complex traits and G × E prediction
Machine learning	Data-driven relationships	Integrates multiple data types	Risk of overfitting	High	Multi-environment and phenomic prediction
Deep learning	Hierarchical feature learning	Handles images and high-dimensional data	Requires large datasets	Very high	Imaging, spectral, and temporal prediction

**Table 3:** Representative applications of genomic prediction for grain yield, grain quality, and disease-resistance traits in rice. The selected studies illustrate the use of diverse rice populations, genome-wide marker datasets, and prediction models for breeding targets that contribute to productivity, market quality, and resistance to major diseases. Prediction results are presented using the terminology and statistical definition applied in each original study. Values should be compared only when the studies used equivalent reference phenotypes, metric calculations, and validation designs.

Breeding Target	Study Material	Prediction Trait	Reported Metric	Validation Design	Main Result	Reference
Grain yield	363 elite irrigated lines; 73,147 GBS markers	Grain yield, plant height, and flowering time	Pearson *r* between GEBV and phenotype, termed accuracy	Structure-aware five-fold cross-validation	Yield prediction accuracy reached 0.31; RR-BLUP performed best	[29]
	111 elite lines; 882 SNPs; 15 environments	Multi-environment prediction of grain yield	Predictive ability: Pearson *r* between predictions and adjusted phenotypes	Random-, selected-, and leave-one-environment-out validation	Predictive ability for grain yield ranged from −0.29 to 0.62 across environments	[19]
	564 stress-tolerant lines crossed with 13 male-sterile lines	Hybrid yield and combining ability	Prediction accuracy calculated from predictive ability and prediction reliability	500 cross-validation replicates with parental representation scenarios	Hybrid genetic-value prediction ranged from 0.490 to 0.822	[39]
Grain quality	404 hybrid lines; 56 K SNP array	Amylose content, alkali value, grain shape, and yield components	Prediction accuracy; calculation not defined	Five-fold cross-validation	Amylose content and alkali value showed high predictive performance	[37]
	417 *indica* rice accessions; 24,502 SNPs	Amylose content and gel consistency	Pearson *r* between GEBVs and reference values	Five-fold cross-validation repeated 30 times	Pearson correlations ranged from 0.832 to 0.836 for amylose content and from 0.708 to 0.724 for gel consistency	[16]
	Rice breeding population evaluated for milling, appearance, cooking, and eating quality	Multi-trait prediction of grain-quality attributes	Pearson *r* between GEBVs and best linear unbiased estimates	Five-fold cross-validation with repeated partitions	Marker covariates and correlated traits improved prediction	[40]
Disease resistance	161 African and 162 United States accessions	Resistance to *Magnaporthe oryzae* isolates	Pearson *r* between GEBVs and disease scores, termed accuracy	Within-population cross-validation and cross-isolate validation	GBLUP accuracy ranged from 0.15 to 0.72	[41]
	Two populations evaluated for leaf and panicle blast	GWAS-weighted GBLUP	Predictive ability: Pearson *r* between GEBVs and best linear unbiased estimates	Stratified five-fold cross-validation repeated 100 times	Marker weighting increased predictive ability by up to 37%	[38]
	1545 recombinant inbred lines from 11 biparental populations	Bayesian prediction of sheath blight resistance	Prediction accuracy; calculation not reported	Random cross-validation; partition details not reported	Prediction accuracy ranged from 0.67 to 0.74.	[42]

**Table 4:** Representative applications of genomic prediction for rice adaptation to drought, salinity, low-input conditions, and other abiotic constraints. The selected studies illustrate the use of multi-environment models, trait-specific markers, recurrent selection, and genomic prediction of physiological and agronomic traits under stress-prone production systems. Prediction results are presented using the terminology and statistical definition applied in each original study. Values should be compared only when the studies used equivalent reference phenotypes, metric calculations, and validation designs.

Breeding Target	Study Material	Prediction Target	Reported Metric	Validation Design	Main Result	Reference
Drought tolerance	280 rainfed lowland accessions; 215,242 SNPs	Flowering time, grain yield, and plant height under non-stress and drought conditions	Predictive ability: Pearson *r* between predictions and phenotypes	Prediction of untested genotypes and prediction of genotypes evaluated in at least one environment; 80:20 split repeated 100 times	Trait-specific markers increased predictive ability by 22%, while multi-environment models improved prediction by 32%	[33]
Water-saving adaptation	284 reference accessions and 97 advanced lines	Flowering time, panicle weight, and nitrogen-balance index under alternate wetting and drying and continuous flooding	Correlation-based predictive ability	Cross-validation and independent progeny validation	Multi-environment models improved prediction by approximately 30%	[34]
Drought breeding	Historical data from 17 breeding years	Grain yield across drought-prone environments	Predictive ability: correlation between predicted and estimated yield	Scenarios for prediction in a new environment, prediction of untested genotypes, and prediction of genotypes evaluated in at least one environment	G × E models improved prediction across target environments	[44]
Salinity tolerance	241 *japonica* accessions; 20,255 SNPs; 393 breeding lines	Growth traits and Na and K concentrations under control and salinity treatments	Predictive ability: Pearson *r* between GEBVs and phenotypes	Prediction of untested genotypes repeated 100 times and independent validation with 41 lines	Predictive ability ranged from 0.25 to 0.64, with validation values reaching 0.66	[30]
Nitrogen-use efficiency	184 upland accessions and 198 advanced lines	Grain yield and nitrogen-use efficiency under low-input conditions	Predictive ability: correlation between predicted and observed phenotypes	Five-fold cross-validation repeated 100 times and across-population validation	Across-population predictive ability ranged from 0.06 to 0.27	[43]
Nitrogen status under water limitation	284 reference accessions and 97 advanced lines	Flowering, panicle weight, and nitrogen-balance index under alternate wetting and drying and continuous flooding	Predictive ability: Pearson *r* between predictions and validation phenotypes	80:20 cross-validation repeated 100 times; progeny validation in 97 lines	Mean predictive ability was 0.31 for the response index and 0.64 for the response slope across traits	[34]
Low grain arsenic accumulation	Bengal and Assam Aus rice panel	Grain arsenic concentration and grain yield	Predictive ability; correlation-based metric	Cross-validation of single-, multi-environment, and multi-trait models	Multi-environment and multi-trait models improved prediction by up to 47% and 61%, respectively	[36]
Upland adaptation	Recurrent GS population evaluated across generations and locations	Multi-generation and multi-location performance	Predictive ability: correlation between predictions and trial-adjusted best linear unbiased estimates	Five scenarios; independent validation in PCT27B at Santa Rosa	Combined generation and location data improved prediction across selection cycles	[45]

## Data Availability

No new datasets were generated or analyzed in this review. All data discussed in this article are available from the cited references.
